# Dietary supplement increases plasma norepinephrine, lipolysis, and metabolic rate in resistance trained men

**DOI:** 10.1186/1550-2783-6-4

**Published:** 2009-01-28

**Authors:** Richard J Bloomer, Kelsey H Fisher-Wellman, Kelley G Hammond, Brian K Schilling, Adrianna A Weber, Bradford J Cole

**Affiliations:** 1 Department of Health and Sport Sciences, University of Memphis, Memphis, TN, USA

## Abstract

**Background:**

Dietary supplements targeting fat loss and increased thermogenesis are prevalent within the sport nutrition/weight loss market. While some isolated ingredients have been reported to be efficacious when used at high dosages, in particular in animal models and/or via intravenous delivery, little objective evidence is available pertaining to the efficacy of a finished product taken by human subjects in oral form. Moreover, many ingredients function as stimulants, leading to increased hemodynamic responses. The purpose of this investigation was to determine the effects of a finished dietary supplement on plasma catecholamine concentration, markers of lipolysis, metabolic rate, and hemodynamics.

**Methods:**

Ten resistance trained men (age = 27 ± 4 yrs; BMI = 25 ± 3 kg· m^-2^; body fat = 9 ± 3%; mean ± SD) ingested a dietary supplement (Meltdown^®^, Vital Pharmaceuticals) or a placebo, in a random order, double blind cross-over design, with one week separating conditions. Fasting blood samples were collected before, and at 30, 60, and 90 minutes post ingestion and were assayed for epinephrine (EPI), norepinephrine (NE), glycerol, and free fatty acids (FFA). Area under the curve (AUC) was calculated for all variables. Gas samples were collected from 30–60 minutes post ingestion for measurement of metabolic rate. Heart rate and blood pressure were recorded at all blood collection times.

**Results:**

AUC was greater for the dietary supplement compared to the placebo for NE (1332 ± 128 pg·mL^-1^·90 min^-1 ^vs. 1003 ± 133 pg·mL^-1^·90 min^-1^; p = 0.03), glycerol (44 ± 3 μg·mL^-1^·90 min^-1 ^vs. 26 ± 2 μg·mL^-1^·90 min^-1^; p < 0.0001), and FFA (1.24 ± 0.17 mmol·L^-1^·90 min^-1 ^vs. 0.88 ± 0.12 mmol·L^-1^·90 min^-1^; p = 0.0003). No difference between conditions was noted for EPI AUC (p > 0.05). For all variables, values were highest at 90 minutes post ingestion. Total kilocalorie expenditure during the 30 minute collection period was 29.6% greater (p = 0.02) for the dietary supplement (35 ± 3 kcal) compared to placebo (27 ± 2 kcal). A condition main effect was noted for systolic blood pressure (p = 0.04), with values increasing from 117 ± 2 mmHg to 123 ± 2 mmHg with the dietary supplement, while remaining unchanged for placebo. No other hemodynamic changes were noted (p > 0.05).

**Conclusion:**

The dietary supplement results in an acute increase in plasma NE and markers of lipolysis, as well as metabolic rate. This occurs without altering hemodynamic variables in a clinically significant manner. Intervention studies to determine the impact of this dietary supplement on weight/fat loss are warranted.

## Background

The prevalence of obesity has grown to epidemic proportions within the United States in recent years, with an estimated 400 million people now being classified as obese [1]. Methods to treat this growing problem traditionally include increased physical activity and modification of dietary intake, as well as surgical, pharmaceutical, and nutritional supplement interventions [2]. Due to the difficulty of maintaining regular physical activity and optimal dietary practices, many individuals seek weight management support in either a pharmaceutical or dietary supplement. Furthermore, due to concern over potential adverse outcomes associated with prescription drug use, many consumers prefer over the counter (OTC) dietary supplements. While some isolated OTC ingredients have been reported to be efficacious in terms of increasing lipolysis, most have only been studied at high dosages, often using animal models or *in vitro *systems, as opposed to human subjects and oral intake [3]. Despite this fact, many dietary supplement manufacturers use such ingredients in their formulations and make claims based on scientific findings that may have little or no relevance to the actual product of sale. This is particularly concerning when the dosage of the "key ingredient" used in many finished products is often far lower than that used in the original research studies. Moreover, many ingredients (e.g., ephedrine) function as stimulants, leading to an undesired and potentially harmful increase in heart rate and blood pressure.

One ingredient that appears to have promise as a dietary aid is yohimbine. Yohim*bine *is a member of the yohimb*ane *family, a large group of indole alkaloids derived from botanical sources. Pharmacologically, yohimbine is well-characterized as an alpha-2-adrenergic receptor antagonist and has been demonstrated to increase lipolysis *in vitro *[3], possibly due to its ability to stimulate a reliable increase in blood norepinephrine (NE); a finding evident in multiple studies involving human subjects receiving single dosages [4-7]. While not as universal a finding, other work has also demonstrated a significant increase in blood epinephrine (EPI) levels with yohimbine intake [7,8]. The increase in NE with acute yohimbine ingestion has been associated with an increase in blood free fatty acid levels [4,5,8], indicating a lipolytic effect of this nutrient. This is likely mediated by the interaction of NE with hormone sensitive lipase (HSL), the rate limiting enzyme in lipolysis [9]. Yohimbine may also aid in lipolysis by acting to improve blood flow [10], and hence, the transport of fatty acids to peripheral tissues to undergo oxidation.

Synephrine (also known as Bitter Orange, Sour Orange, and Seville Orange) has been suggested as an effective dietary aid, as this trace endogenous bioamine activates beta-3 receptors and may result in lipolysis and appetite suppression [11]. Since April 12, 2004 when the Food and Drug Administration banned the sale of dietary supplements containing ephedrine alkaloids, interest in synephrine has risen sharply. In fact, many ephedrine-free products currently being sold contain synephrine as an active ingredient.

Caffeine is a central nervous system stimulant, technically classified as a methylxanthine, which has a temporary effect on increasing lipolysis and thermogenesis [12,13]. This is likely due to its action on the "second messenger system" known as 3', 5'-cyclic adenosine monophosphate (cAMP), which is a crucial component in fatty acid metabolism where it functions to activate cAMP dependent protein kinase [14]. Caffeine has the ability to both decrease the breakdown of cAMP, as well as increase cAMP production via beta-adrenergic receptor independent and dependent mechanisms, respectively [12]. Caffeine is also an adenosine antagonist, capable of blocking the inhibitory effects of adenosine on further NE release, ultimately resulting in an increased or sustained level of NE in the circulation [15]. As such, caffeine is popular as a dietary weight/fat loss aid.

**Figure 1:**
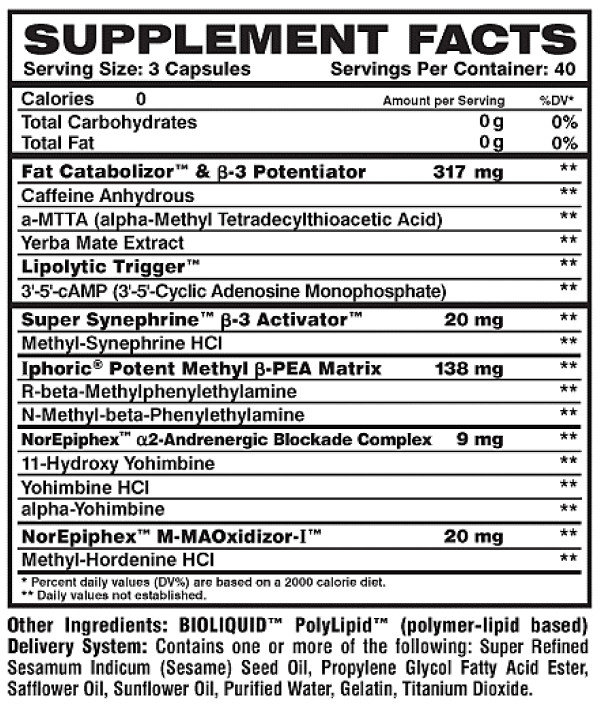
**Label description of Meltdown^®^**.

It is unknown what the potential effects of the above combination of ingredients would be on blood catecholamines and markers of lipolysis. Recently, these ingredients (in addition to other ingredients as presented in Figure 1) have been combined for delivery as one convenient capsule (Meltdown^®^; Vital Pharmaceuticals, Inc.). Two initial studies using this product have noted an increase in subjects' resting [16,17] and post exercise [17] metabolic rate when compared to placebo. However, neither of these studies included blood measurement of catecholamines or markers of lipolysis. Therefore, the interpretation of findings is limited. It was our purpose in the proposed research to extend these findings by studying the impact of this dietary supplement on blood catecholamine levels, markers of lipolysis, metabolic rate, and hemodynamics in human subjects. Using a double blind, randomized, crossover design, we hypothesized that the dietary supplement would result in an increase in NE, markers of lipolysis, and metabolic rate in our sample of resistance trained men, in comparison to a placebo.

## Methods

### Subjects

Ten healthy, resistance trained men participated in this investigation. Subjects completed a medical history and physical activity questionnaire to determine eligibility. No subject was a smoker or had diagnosed metabolic or cardiovascular disease. Subjects were considered to be well-trained and performed resistance exercise for 4.6 ± 2.2 hrs per week for 8.4 ± 6.6 yrs. Descriptive characteristics are provided in Table 1. Subjects were instructed not to deviate from their current training regimen during the course of the study with the exception of refraining from exercise for the 48 hours prior to each testing day. All experimental procedures were performed in accordance with the Helsinki Declaration. The University of Memphis Human Subjects Committee approved all experimental procedures. All subjects provided both verbal and written consent prior to participating in this study.

**Table 1:** Descriptive characteristics of 10 exercise trained men.

Variable	Value
Age (yrs)	27 ± 4
Height (cm)	175 ± 7
Weight (kg)	77 ± 11
Body mass index (kg·m^-2^)	25 ± 3
Body fat (%)*	9 ± 3
Years Resistance Exercise	8 ± 7
Hours/wk Resistance Exercise	5 ± 2

Data are mean ± SD.

* Determined from 7-site skinfold analysis use Lange calipers and Siri equation

### Conditions and Testing

The dietary supplement used in this investigation (Meltdown^®^, Vital Pharmaceuticals, Inc., Davie, FL) included yohimbine, caffeine, and synephrine as the primary active ingredients. Please see Figure 1 for a description of the dietary supplement. All capsules used in this investigation were from the same bottle and produced in accordance with Good Manufacturing Practices (GMPs). Prior to production, all raw materials were tested for ingredient potency and the finished product was verified for label claims. Subjects consumed three capsules of the dietary supplement or an identical looking placebo (corn starch, microcrystalline cellulose, super refined sesame oil, propylene glycol fatty acid ester, safflower oil, sunflower oil) in a double blind, cross-over design. No food was allowed until testing was completed, although water was allowed ad libitum and matched for both days of testing (mean intake = 500 mL).

Subjects reported to the laboratory in a fasted state (> 8 hours), without caffeine consumption during the past 8 hours. All testing was done between 0600–1000 hours. Following a 10 minute quiet rest period, a baseline blood sample was obtained (0 min). Subjects then ingested either the supplement or placebo, in the presence of an investigator. Subjects remained inactive during the entire 90 minute test period. At 30 minutes post ingestion, a second blood sample was taken (30 min). A measurement of resting metabolic rate, using indirect calorimetry, was then started and continued for 30 minutes. Subjects were positioned in a seated posture and gas analysis was performed with breath-by-breath collection using a SensorMedics Vmax 229 metabolic system (Yorba Linda, CA) and facemask. Subjects were familiarized to the facemask prior to the collection period, instructed to simply breathe normally, and to remain quiet and awake during the 30 minute measurement period. Subjects were allowed to read during the collection period. All gas collection took place in a temperature and humidity controlled laboratory, and both the flow sensor and gas analyzers were calibrated prior to data collection. Total oxygen consumption (L·min^-1^) was determined and total kilocalorie expenditure was estimated from this value. Respiratory exchange ratio was also determined from gas collection (CO_2_/O_2_), and used as a crude measure of substrate utilization. At the end of the 30 min collection period, a third blood sample was taken (60 min). A final blood sample was taken at 90 min (90 min). Measurements of heart rate (via heart rate monitor) and blood pressure (via auscultation) were taken immediately prior to each blood sample, in a seated position. Procedures were identical for both test sessions (supplement and placebo).

### Blood Processing and Biochemistry

A total of four venous blood samples (7 mL per draw) were taken from subjects' forearm via needle and Vacutainer^® ^by a trained phlebotomist. Following collection, blood samples were immediately processed in a refrigerated centrifuge in order to obtain plasma (4°C for 15 min at 2000 × g). Plasma samples were stored in multiple aliquots at -80°C. All assays were performed within two months of sample collection, in duplicate, and on first thaw. NE and EPI were determined using an enzyme linked immunosorbent assay (2-CAT ELISA, BA 10–1500; Rocky Mountain Diagnostics) following the instructions of the manufacturer (Labor Diagnostika Nord GmbH & Co. KG). In this competitive ELISA, NE and EPI are extracted by using a cis-diol-specific affinity gel, acylated, and then derivitized enzymatically. The coefficient of variation (CV) for NE and EPI was 9.8% and 6.9%, respectively. Glycerol was determined using the Free Glycerol Determination Kit (FG0100) and Glycerol Standard (G7793), following the instructions of the manufacturer (Sigma Aldrich). The CV for glycerol was 7.8%. Free fatty acids were determined using the Free Fatty Acid Quantification Kit (K612-100) following the instructions of the manufacturer (BioVision). The CV for FFA was 9.2%.

### Diet and Physical Activity

During the 24 hours before each test day, subjects consumed prepackaged meal replacement drinks and bars provided by the project sponsor. These contained a mix of protein, carbohydrate, and fat. Subjects were given 3 shakes and 3 bars and instructed to consume as many as they desired, with no other food or calorie containing drinks. The amount consumed during the day preceding the initial test day was mimicked during the day preceding the second test day. The average intake of subjects was a combination of 5 shakes/bars. This provided approximately 2000 kilocalories. Regarding physical activity, subjects were asked to avoid strenuous exercise during the 48 hours preceding the test days.

### Statistical Analysis

Area under the curve (AUC) was calculated for each biochemical variable for both conditions using the trapezoidal method (AUC_G_) as described in detail by Pruessner et al. [18]. Statistical comparisons for biochemical (AUC_G_) and metabolic data were made between conditions using t-tests. Biochemical data, in addition to heart rate and blood pressure data, were also compared using a 2 (condition) × 4 (time) analysis of variance (ANOVA). Tukey's post hoc tests were used where appropriate. All analyses were performed using JMP statistical software (version 4.0.3, SAS Institute, Cary, NC). Statistical significance was set at P ≤ 0.05. The data are presented as mean ± SEM, except for subject descriptive characteristics (mean ± SD).

## Results

**Figure 2:**
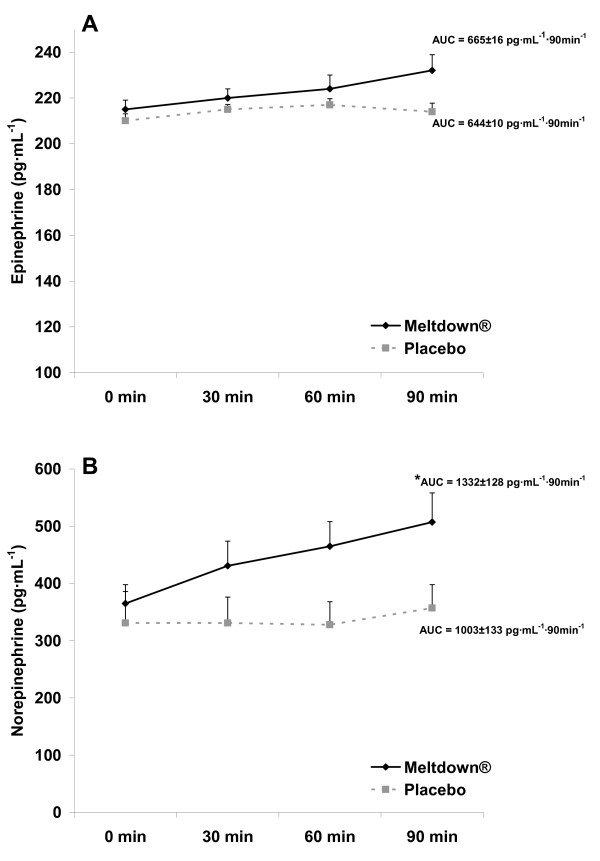
**Plasma epinephrine (A) and norepinephrine (B) data for 10 men consuming Meltdown^® ^and placebo in a randomized cross-over design**. Data are mean ± SEM. * Greater norepinephrine AUC for Meltdown^® ^compared to placebo (p = 0.03).

**Figure 3:**
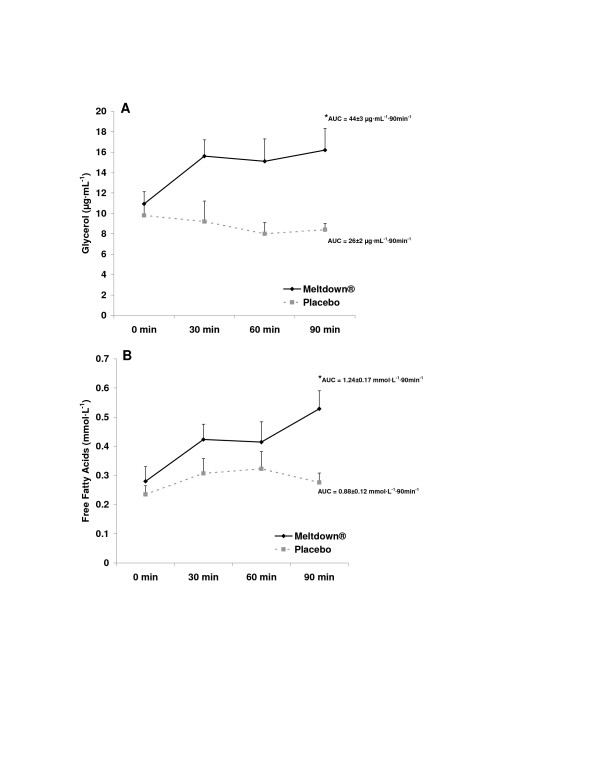
**Plasma glycerol (A) and free fatty acid (B) data for 10 men consuming Meltdown^® ^and placebo in a randomized cross-over design**. Data are mean ± SEM. * Greater glycerol (p < 0.0001) and FFA (p = 0.0003) AUC for Meltdown^® ^compared to placebo.

**Figure 4:**
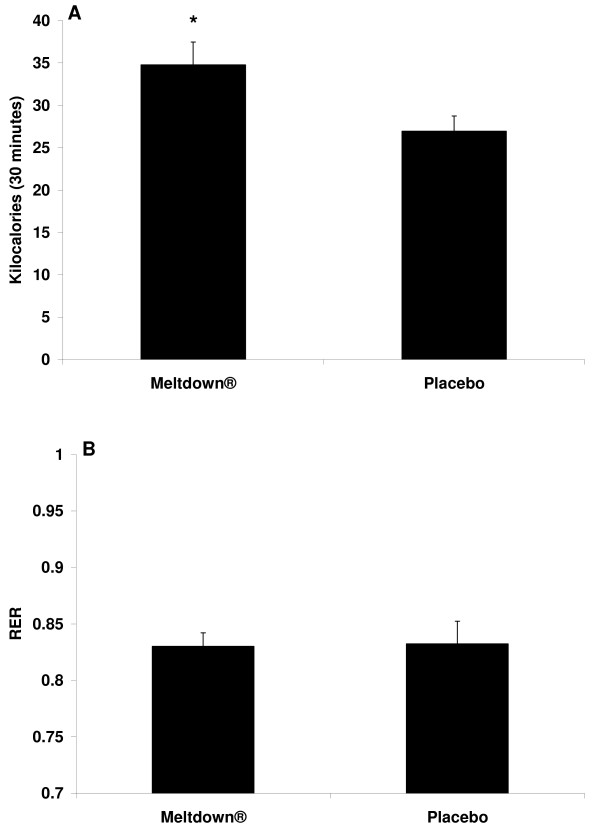
**Total kilocalories (A) and mean respiratory exchange ratio (B) data for 10 men consuming Meltdown^® ^and placebo in a randomized cross-over design**. Data are mean ± SEM. * Greater total kilocalories for Meltdown^® ^compared to placebo (p = 0.02).

All subjects successfully completed all aspects of the study. AUC was greater for the dietary supplement compared to the placebo for NE (Figure 2B; p = 0.03), glycerol (Figure 3A; p < 0.0001), and FFA (Figure 3B; p = 0.0003). No difference was noted between conditions for EPI (Figure 2A; p > 0.05). For all variables, values were highest at 90 minutes post ingestion. When performing the 2 × 4 ANOVA for biochemical variables, a condition main effect was noted for NE (p < 0.0001), with no time effect (p = 0.13) or interaction noted (p = 0.25). A condition main effect was noted for EPI (p = 0.04), with no time effect (p = 0.09) or interaction noted (p = 0.36). An interaction was noted for glycerol (p = 0.0006), with values higher for supplement compared to placebo at 30, 60, and 90 minutes post ingestion (p < 0.05), and higher for supplement at all times post ingestion compared to pre ingestion (p < 0.05). A condition main effect was noted for FFA (p = 0.0003), with no time effect (p = 0.08) or interaction noted (p = 0.32). Total kilocalorie expenditure during the 30 minute collection period was 29.6% greater (p = 0.02) for the dietary supplement compared to placebo (Figure 4A). No difference was noted between conditions for respiratory exchange ratio (Figure 4B; p > 0.05). A condition main effect was noted for systolic blood pressure (p = 0.04), with values increasing from 117 ± 2 mmHg to 123 ± 2 mmHg with the dietary supplement, while remaining unchanged for placebo. No other hemodynamic changes were noted (p > 0.05). Hemodynamic data are presented in Table 2.

**Table 2:** Hemodynamic data for 10 men consuming Meltdown^® ^and placebo in a randomized cross-over design.

Variable	0 min	30 min	60 min	90 min
Heart rate (bpm) *Meltdown^®^*	59 ± 3	63 ± 2	62 ± 2	63 ± 2
Heart rate (bpm) *Placebo*	59 ± 3	60 ± 3	62 ± 3	60 ± 3
Systolic Blood Pressure (mmHg) Meltdown^® ^*	117 ± 2	122 ± 3	123 ± 2	122 ± 3
Systolic Blood Pressure (mmHg) *Placebo*	118 ± 2	118 ± 2	117 ± 1	116 ± 1
Diastolic Blood Pressure (mmHg) *Meltdown^®^*	72 ± 1	71 ± 2	72 ± 2	70 ± 2
Diastolic Blood Pressure (mmHg) *Placebo*	72 ± 1	72 ± 2	71 ± 1	71 ± 1

Data are mean ± SEM.

*Condition effect; higher systolic blood pressure for Meltdown^® ^compared to placebo (p = 0.04).

No other statistically significant effects noted (p > 0.05).

## Discussion

Data from the present investigation indicate that the dietary supplement Meltdown^®^, ingested at the exact dosage as recommended by the manufacturer, results in an acute increase in plasma NE, glycerol and FFA (when measured using AUC; in addition to a condition main effect for EPI when measured using ANOVA), as well as an increase in metabolic rate. This occurs despite only a mild increase in heart rate and systolic blood pressure, with no increase in diastolic blood pressure. Although metabolic rate was higher for Meltdown^® ^compared to placebo, it should be noted that the typical day-to-day variance in this measure is estimated at 4–6% [19]. Hence, this should be considered when interpreting our findings.

Although it is impossible to determine which of the active ingredients contained with this and other finished products are actually responsible for the observed effects, it is likely that the present findings are due to the three primary ingredients in Meltdown^®^; yohimbine, caffeine, and synephrine. Based on our findings of minimal hemodynamic changes, coupled with the significant increase in NE, we believe that yohimbine may be the most important component to this supplement.

The process of fatty acid oxidation involves the complex interplay between HSL, the specific hormones acting to stimulate HSL, and the receptors that bind to these hormones in order for them to exert their effect [9]. Although many hormones may be involved in fatty acid metabolism (e.g., growth hormone, thyroid hormone, ACTH, cortisol), the catecholamines EPI and NE appear paramount [9]. These interact with both beta adrenergic receptors (EPI and NE), as well as alpha-adrenergic receptors (NE). Depending on which receptors are activated, lipolysis can be either stimulated (beta) or inhibited (alpha), with optimal HSL activity observed in the presence of low insulin levels.

While yohimbine itself has been reported in several studies to increase blood NE [4-7], NE is not selective in its binding. That is, while it can bind beta receptors (1, 2, and 3 sub-class), it also binds alpha receptors (1 and 2 sub-class) [20]. Therefore, in a way, NE may both "stimulate" and "inhibit" fatty acid mobilization. Specifically, activation of alpha 2 receptors inhibits further release of NE, allowing NE to act as its own negative feedback signal. Because yohimbine is a selective alpha-2-adrenergic receptor *antagonist*, it can function to impair the negative feedback loop specific to NE. This effect, coupled with the stimulatory effect of yohimbine on NE release, allows for a net increase in circulating NE. This was clearly demonstrated in the present investigation (Figure 2B). This occurred despite the relatively low dosage of yohimbine provided (9 mg) compared to other studies using dosages equal to 2–5 times this amount [4-7]. It is possible that the form of yohimbine used in the dietary supplement could be responsible for the significant increase in NE, as a combination of yohimbine HCl, alpha-yohimbine, and 11-hydroxy yohimbine make up the total yohimbine complex provided in Meltdown^®^.

Although HSL may be ultimately stimulated by the increase in EPI and NE, it is the initial binding of the catecholamines to beta receptors that begins the secondary intracellular activation of adenylyl cyclase [21]. Activation of adenylyl cyclase results in an increased production of cAMP [14], which in turn leads to the activation of a cAMP dependent protein kinase (PKA) [22]. It is PKA that ultimately activates HSL leading to triglyceride breakdown and subsequent release of glycerol and FFA into the circulation.

Caffeine possesses lipolytic/thermogenic effects due to its ability to both decrease the breakdown of cAMP as well as increase cAMP production via beta-adrenergic receptor independent and dependent mechanisms, respectively [12]. The independent effects are due to caffeine's ability to directly inhibit cAMP degradation, by inhibiting the cyclic nucleotide phosphodiesterase [23] and blocking adenosine receptors (anti-lipolytic agent receptors). The direct effect results from an increase in catecholamine release following caffeine ingestion, which may be secondary to the previously described adenosine inhibition [12]. The potential role of synephrine as a lipolytic agent is also specific to its ability to interact with beta receptors (3 sub-class), thereby promoting lipolysis via the above described cAMP dependent mechanism [24].

In addition to yohimbine, caffeine, and synephrine, several other ingredients are included within Meltdown^®^. These include the amphetamine-like/thyroid stimulating agent phenylethylamine (PEA), which has been reported to cause a significant reduction in 24 hour food intake, and a dose dependent reduction in body weight gain in rats [25]. This may be due partly to the effect of PEA on stimulating blood catecholamine levels and inhibiting their reuptake [26]. The monoamine oxidase inhibitor methyl hordidine is also contained within this supplement. Hordinine is structurally similar to EPI and has been shown to liberate NE from its storage site, in addition to inhibiting NE metabolism [27]. The lipolytic agent methyl tetradecylthioacetic acid is also included. It is known to stimulate beta oxidation [28] and is clearly involved in lipid transport and utilization [29]. Finally, the satiety hormone cholecystokinin (CCK-8) may have an influence on food intake if provided over a prolonged period of time.

Collectively, the above ingredients appear to represent a substantial list of potentially effective lipolytic agents. While it is possible that these additional ingredients may have contributed to the overall effectiveness of the dietary supplement in regards to our findings of increased lipolysis and metabolic rate, based on the relatively low dosages provided (in comparison to those used in prior investigations where these ingredients have been studied in isolation), it is difficult to state with certainty that their contribution was significant.

It is important to note that our findings for all blood variables following intake of the dietary supplement were highest at the 90 minute post ingestion mark. It is indeed possible that further increases may have been observed at times distant to this. Further study to determine the time course of increased lipolysis is warranted. Based on the work of Hoffman et al. [16] who noted an increase in metabolic rate during hours one, two, and three following ingestion of this dietary supplement, it is likely that the corresponding blood variables would also remain elevated during this time. If so, the potential for increased fat mobilization is apparent. More importantly, if coupled with acute bouts of exercise, fat "burning" may be increased significantly during this period of time, potentially resulting in decreased body weight/body fat. Of course, longer term intervention studies are needed to test this hypothesis.

## Conclusion

In conclusion, we report that the finished product Meltdown^®^, ingested at the exact dosage as recommended by the manufacturer, results in an acute increase in plasma NE, glycerol, and FFA (measured using AUC), EPI (measured using ANOVA), as well as metabolic rate. This occurs despite a minimal increase in heart rate and systolic blood pressure. Our findings are specific to a sample of young, healthy, and lean resistance trained men. Further study is needed to determine if similar or more pronounced findings are observed in a sample of overweight/sedentary men and women, who often respond to a greater extent to such treatment. Longer term studies are also needed to determine if the lipolytic effects of this supplement extend beyond 90 minutes post ingestion. Finally, intervention studies are warranted to determine the impact of this dietary supplement on weight/fat loss.

## Competing interests

This study was financially supported by Vital Pharmaceuticals, Inc. Although the authors or the University of Memphis do not directly endorse the dietary supplement, the lead author (RJB) has been involved in scientific writing for Vital Pharmaceuticals, Inc.

## Authors' contributions

RJB was responsible for the study design, biochemical work, statistical analyses, and manuscript preparation; KHFW, KGH, AAW, BJC were responsible for data collection, blood collection and processing; BKS was responsible for the study design and manuscript preparation. All authors read and approved of the final manuscript.

## References

[B1] Consitt LA, Bell JA, Houmard JA (2009). Intramuscular lipid metabolism, insulin action, and obesity. IUBMB Life.

[B2] Crowley VE (2008). Overview of human obesity and central mechanisms regulating energy homeostasis. Ann Clin Biochem.

[B3] Caruso MK, Roberts AT, Bissoon L, Self KS, Guillot TS, Greenway FL (2008). An evaluation of mesotherapy solutions for inducing lipolysis and treating cellulite. J Plast Reconstr Aesthet Surg.

[B4] Barbe P, Galitzky J, Riviere D, Senard JM, Lafontan M, Garrigues M, Berlan M (1993). Effects of physiological and pharmacological variation of sympathetic nervous system activity on plasma non-esterified fatty acid concentrations in man. Br J Clin Pharmacol.

[B5] Galitzky J, Taouis M, Berlan M, Rivière D, Garrigues M, Lafontan M (1988). Alpha 2-antagonist compounds and lipid mobilization: evidence for a lipid mobilizing effect of oral yohimbine in healthy male volunteers. Eur J Clin Invest.

[B6] Lenders JW, Golczynska A, Goldstein DS (1995). Glucocorticoids, sympathetic activity, and presynaptic alpha 2-adrenoceptor function in humans. J Clin Endocrinol Metab.

[B7] Petrie EC, Peskind ER, Dobie DJ, Veith RC, Raskind MA (2000). Increased plasma norepinephrine response to yohimbine in elderly men. J Gerontol A Biol Sci Med Sci.

[B8] Valet P, Taouis M, Tran MA, Montastruc P, Lafontan M, Berlan M (1989). Lipomobilizing effects of procaterol and yohimbine in the conscious dog: comparison of endocrinological, metabolic and cardiovascular effects. Br J Pharmacol.

[B9] Duncan RE, Ahmadian M, Jaworski K, Sarkadi-Nagy E, Sul HS (2007). Regulation of lipolysis in adipocytes. Annu Rev Nutr.

[B10] Wray DW, Raven PB, Sander M (2008). Diminished baroreflex-induced vasoconstriction following alpha-2 adrenergic receptor blockade in humans. Auton Neurosci.

[B11] Fugh-Berman A, Myers A (2004). Citrus aurantium, an ingredient of dietary supplements marketed for weight loss: current status of clinical and basic research. Exp Biol Med.

[B12] Acheson KJ, Gremaud G, Meirim I (2004). Metabolic effects of caffeine in humans: lipid oxidation or futile cycling?. Am J Clin Nutr.

[B13] Graham TE (2001). Caffeine and exercise: metabolism, endurance and performance. Sports Med.

[B14] Collins S, Cao W, Robidoux J (2004). Learning new tricks from old dogs: beta-adrenergic receptors teach new lessons on firing up adipose tissue metabolism. Mol Endocrinol.

[B15] Fisone G, Borgkvist A, Usiello A (2004). Caffeine as a psychomotor stimulant: mechanism of action. Cell Mol Life Sci.

[B16] Hoffman J, Kang J, Ratamess N, Rashti S, Tranchina C, Kelly N, Faigenbaum A (2008). Thermogenic effect of an acute ingestion of a weight loss supplement. J Int Soc Sports Nutr.

[B17] Jitomir J, Nassar E, Culbertson J, Moreillon J, Cooke M, Buford T, Hudson G, Willoughby D (2008). VPX Meltdown^® ^significantly increases energy expenditure and fat oxidation without effecting hemodynamic variables in a randomized, double-blind, cross-over clinical research trial. J Int Soc Sports Nutr.

[B18] Pruessner JC, Kirschbaum C, Meinlschmid G, Hellhammer DH (2003). Two formulas for computation of the area under the curve represent measures of total hormone concentration versus time-dependent change. Psychoneuroendocrinology.

[B19] Roffey DM, Byrne NM, Hills AP (2006). Day-to-day variance in measurement of resting metabolic rate using ventilated-hood and mouthpiece & nose-clip indirect calorimetry systems. J Parenter Enteral Nutr.

[B20] Morales A (2000). Yohimbine in erectile dysfunction: the facts. Int J Impot Res.

[B21] Gonzalez-Yanes C, Sanchez-Margalet V (2006). Signaling mechanisms regulating lipolysis. Cell Signal.

[B22] Taylor SS, Kim C, Cheng CY, Brown SH, Wu J, Kannan N (2008). Signaling through cAMP and cAMP-dependent protein kinase: diverse strategies for drug design. Biochim Biophys Acta.

[B23] Butcher RW, CE Baird, EW Sutherland (1968). Effects of lipolytic and antilipolytic substances on adenosine 3',5'-monophosphate levels in isolated fat cells. J Biol Chem.

[B24] Carpéné C, Galitzky J, Fontana E, Atgié C, Lafontan M, Berlan M (1999). Selective activation of beta3-adrenoceptors by octopamine: comparative studies in mammalian fat cells. Naunyn Schmiedebergs Arch Pharmacol.

[B25] Dourish CT, Boulton AA (1981). The effects of acute and chronic administration of beta-phenylethylamine on food intake and body weight in rats. Prog Neuropsychopharmacol.

[B26] Paterson IA, Juorio AV, Boulton AA (1990). 2-Phenylethylamine: a modulator of catecholamine transmission in the mammalian central nervous system?. J Neurochem.

[B27] Hapke HJ, W Strathmann (1995). Pharmacological effects of hordenine. Dtsch Tierarztl Wochenschr.

[B28] Berge RK, Hvattum E (1994). Impact of cytochrome P450 system on lipoprotein metabolism. Effect of abnormal fatty acids (3-thia fatty acids). Pharmacol Ther.

[B29] Berge RK, Tronstad KJ, Berge K, Rost TH, Wergedahl H, Gudbrandsen OA, Skorve J (2005). The metabolic syndrome and the hepatic fatty acid drainage hypothesis. Biochimie.

