# Dietary supplement increases plasma norepinephrine, lipolysis, and metabolic rate in resistance trained men

**DOI:** 10.1186/1550-2783-6-10

**Published:** 2009-04-17

**Authors:** Richard J Bloomer, Kelsey H Fisher-Wellman, Kelley G Hammond, Brian K Schilling, Adrianna A Weber, Bradford J Cole

**Affiliations:** 1 Department of Health and Sport Sciences, University of Memphis, Memphis, TN, USA

## Abstract

Correction to Richard J Bloomer, Kelsey H Fisher-Wellman, Kelley G Hammond, Brian K Schilling, Adrianna A Weber and Bradford J Cole: Dietary supplement increases plasma norepinephrine, lipolysis, and metabolic rate in resistance trained men. *Journal of the International Society of Sports Nutrition *2009, 6: 4

## Correction

Following publication of our recent article [1], we noticed an error in Figure 2 A. The units of measure on the y-axis should range from 0 to 100 pg ml^-1 ^rather than 100–240 pg ml^-1 ^as stated in the original article.

The corrected Figure 2 is presented here (Figure 1). The results and conclusions of this article remain unchanged.

**Figure 1:**
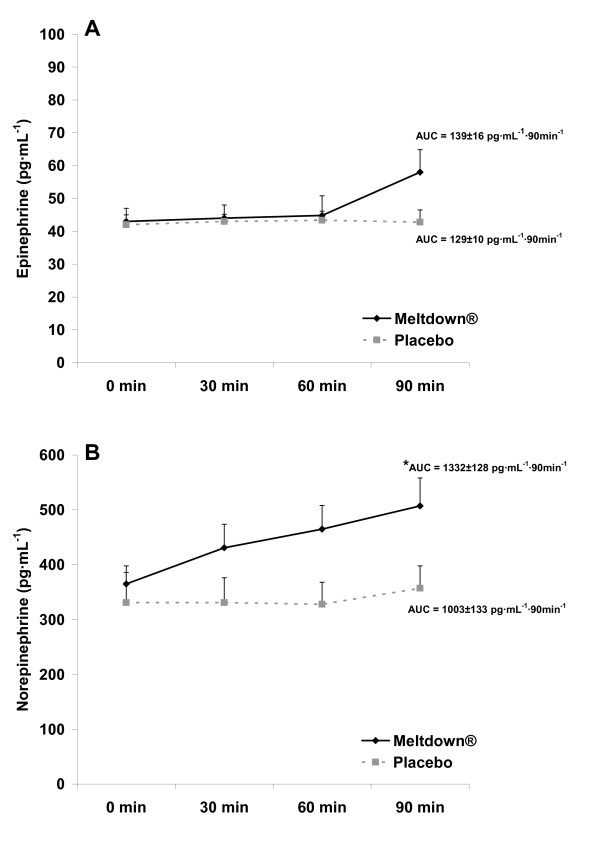
**Plasma epinephrine (A) and norepinephrine (B) data for 10 men consuming Meltdown^® ^and placebo in a randomized cross-over design**. Data are mean ± SEM. * Greater norepinephrine AUC for Meltdown^® ^compared to placebo (p = 0.03).
